# Galactosaminoglycan Function and Oligosaccharide Structure Determination

**DOI:** 10.1100/tsw.2007.63

**Published:** 2007-02-19

**Authors:** Daniela G. Seidler, Jasna Peter-Katalinic, Alina D. Zamfir

**Affiliations:** ^1^ Department of Physiological Chemistry and Pathobiochemistry, UKM, University of Münster, D-48149, Münster, Germany; ^2^ Institute for Medical Physics and Biophysics, Biomedical Analysis Department, UKM, University of Münster, D-48149, Münster, Germany; ^3^ Department of Chemistry and Biology, University of Arad, RO-310139, Arad, Romania; ^4^ Mass Spectrometry Division, National Institute for Research and Development in Electrochemistry and Condensed Matter, RO-300224, Timisoara, Romania

**Keywords:** dermatan sulfate, oligosaccharide structural analysis, electrospray mass spectrometry, MS/MS sequencing

## Abstract

This review will discuss the importance of sequencing long chondroitin sulfate and dermatan sulfate chains specifically derived from decorin. Decorin is a member of the small leucine-rich repeat proteoglycans and ubiquitously expressed primarily in the skin. Sequence information and diverse function of glycosaminoglycans is further influenced by variable expression through the core protein indicating the importance to analyse glycosaminoglycans from specific proteoglycans.

## INTRODUCTION

Glycosaminoglycans are linear polysaccharides exhibiting a wide range of biological function. The primary structure of several glycosaminoglycans is organized macroscopically by segregation into alternating blocks of specific sulfation patterns and microscopically by formation of oligosaccharide sequences with specific binding functions. The biological importance of the fine structures of dermatan sulfate has been demonstrated by the finding that particular sulfated oligosaccharide sequence within these glycosaminoglycans can confer growth factor or anticoagulant activity.

### Galactosaminoglycans and Function

Proteoglycans are a group of proteins covalently linked with sulfated glycosaminoglycan (GAG) chains. The GAG can be either heparan sulfate or chondroitin/dermatan sulfate (CS/DS) or keratan sulfate. CS/DS is a sulfated galactosaminoglycan attached to core proteins, like decorin and biglycan[1]. In humans, aberrant GAG substitution of decorin was observed in Ehlers-Danlos syndrome (EDS) patients who carried mutations in the *B4GALT7* gene encoding galactosyltransferase I (ß4GalT-7; E.C. 2.4.1.133), which catalyses the transfer of galactose to the xylose residue forming the linkage region of proteoglycans[2,3]. The patient's fibroblasts displayed reduced ß4GalT-7 activity and synthesized ~50% decorin protein core and monoglycanated biglycan[4]. Typical EDS features are craniofacial appearance and skeletal abnormalities and a delay in wound healing. In animal models, the loss of decorin[5] and biglycan[6, 7] has profound effects on bone, and skin, which are the major target tissues of EDS in humans. These examples demonstrate the importance of understanding the function of decorin and its galactosaminoglycan chain.

GAG structures are based on disaccharide repeats and there are four different ones. Each is classified by a particular repeating disaccharide, like CS which is composed of glucuronic acid-(β-1, 4)-N-acetyl-galactosamine-(α-1, 4). During the biosynthesis in the Golgi, even when the GAG is still being elongated, the sugar moieties are subjected to a set of modifications. Depending on tissue, the glucuronic acid (GlcA) residues can be epimerized to L-iduronic acid (L-IdoA), the GAG chains are then called DS[1]. Among the GAGs, DS, heparin and heparan sulfate show considerable conformational flexibility due to the presence of L-IdoA residues, which change easily between chair and skew conformations[8]. The chondroitin C5-epimerase (EC 5.1.3.19) was the last missing enzyme involved in the biosynthesis of DS[9]. Interestingly, the chondroitin C5-epimerase was first described as SART2, a protein of unknown function, overexpressed in cancer cells[10]. Furthermore, GalNAc and L-IdoA can be differently sulfated. This lead to a functional microheterogeneity of CS/DS chains. The epimerisation and sulfation is not random but rather reflects a controlled enzymatic system where the protein is transported to compartments differing in enzyme activity[11] to encrypt functional information into the GAG moiety. The biological function of the GAG chains is extensively studied for heparan sulfates[12]. However, a growing body of evidence has identified CS/DS, as playing a role in a large number of biological functions as coagulation, immune response and cell growth[13].

Much less is known about potentially active domains of CS/DS. Interactions with heparin cofactor II and protein C inhibitor have been described[14,15]. DS may interact in place of heparin or heparan sulfate with matrix proteins, like fibronectin[16], cell membrane receptors and platelet factor 4[17]. Another important aspect of CS/DS was reported in studies on tumor metastasis. There are indications that CS/DS may regulate angiogenesis and melanoma cell invasion and proliferation[18]. Recently, Kawashima and co-workers[19] reported that versican, a large CS/DS proteoglycan, interacts through its CS/DS chains with the adhesion molecules L- and P-selectin and that this interplay is sulfation-dependent and requires oversulfated domains of CS/DS.

FGF-2 is known to bind to the GAGs heparin sulfate and heparin on cells and in the extracellular matrix to be stored and protected from proteolytic degradation[20]. A model has been proposed in which cell surface heparin sulfate function as low affinity co-receptor for FGF-2 and present it to the high affinity signaling receptor[21,22]. During wound healing cell surface and extracellular proteoglycans are found in wound fluid and the FGF-2 activating GAG derived from wound fluid was DS[23] These GAGs might be linked to decorin or biglycan, two DS-proteoglycans which play a crucial role in wound healing[24]. However, the nature of the FGF-2 binding domain within DS is not known yet, as is the nature of the core protein where the interacting DS chain is derived from. Furthermore, DS plays a role in the signalling behavior of fibroblast growth factor-7, hepatocyte growth factor/scatter factor, and also regulates WISP-1 function[25,26,27].

The polysaccharide structure of several GAGs is characterized by the appearance of alternating blocks with characteristic epimerisation and sulfation patterns, thereby probably forming oligosaccharide sequences with specific binding functions.

## Structural Analysis of Oligosaccharides Derived from Galactosaminoglycans

For structure investigation of CS/DS glycoforms, development of high performance specific methods was lately required, among which, mass spectrometry (MS) contributed an essential progress to the field[28]. MS with electrospray (ESI) became nowadays the most valuable technique for structural and quantitative

CS/DS investigation because it uniquely offers the combination of analytical versatility, sensitivity, and precision.

Prior to ESI introduction difficulties encountered in the ionization of CS/DS mixtures limited for long time the MS potential for their structural elucidation. The most severe problems in this regard were related to the impossibility to obtain high ionization yield for long chains, hamper unspecific *in-source* decomposition of the labile sulfate group, generate the multiply charged ions of high molecular weight species and finally distinguish the isobaric structures. Under appropriate optimized conditions, and negative ion mode detection settings, intact negative ions can be formed and transferred into the MS without a significant in-source fragmentation, which would primordially lead to desulfation and induction of spectrum/interpretation artifacts. Another advantage of the method is that the majority of modern mass spectrometers working in conjunction with ESI exhibit the tandem MS facility, which gives the possibility to sequence the precursor ion and thus obtain straightforward information upon the repeating disaccharide unit, number of sulfate groups and their precise location along the CS/DS chain and/or within a certain monomer ring[29,30]. In the case of chain depolymerization using lyases complex CS/DS mixtures containing intact chains of variable length are formed. In ESI-MS, because of chain constitution, the monoisotopic peaks corresponding to charge states that equal ½ the number of the repeating units are observed at the same *m*/*z* value. This isobaric peak overlapping results in another specific challenges of ESI-MS of GAGs in general and CS/DS in particular since it makes the screening spectra difficult to interpret and jeopardizes the isolation of the precursor ion for tandem MS. For these reasons, the screening of the complex DS mixtures requires the combination of MS with an efficient separation technique properly optimized for this type of analysis. From this point of view, ESI is also advantageous as it allows for on-line coupling of MS with chromatographic separation methods such as size exclusion chromatography (SEC)[31], high performance liquid chromatography[32] and capillary electrophoresis (CE)[33].

Novel insights into the domain structure of the hybrid CS/DS chains obtained from bovine aorta by enzymatic depolymerization using chondroitin B lyase, which is the characteristic of oligosaccharides representing defined hybrid molecules bearing a single DS disaccharide unit at the non-reducing end, linked to a variable number of CS disaccharide units at the reducing terminal, were recently obtained using an analytical approach based on CE in conjunction with negative ESI-quadrupole time-of-flight tandem mass spectrometry (QTOF-MS/MS)[34]. By CE fractionation followed by ESI-MS detection, several oligosaccharides containing one $SO_3^-$ per disaccharide were identified. The species were all detected bearing one double bond per molecule, which clearly indicated that contain L-IdoA building block formed by elimination during the enzymatic cleavage of the polysaccharide chain. The ion corresponding to a composition of Δ-unsaturated 4,5-Δ-IdoAGalNAc[GlcAGalNAc]$_{3(}$4S) octasaccharide, has been selected as a precursor for sequencing in a tandem MS experiment. Beside ions indicative of a regularly sulfated pattern, two detected oversulfated fragments showed that in the investigated octasaccharide chain one disaccharide unit might be disulfated. Moreover, it could be postulated that the ions might arise from either the same sequence species, or document even two different locations of the oversulfation. Additional significant structural aspect revealed by this analysis is the determination of the sulfate linkage site. According to the relative intensity of typical cleavages determined by tandem MS, it was clearly established that in the disaccharide units constituting the octasaccharide CS/DS of the bovine aorta, the GalNAc is sulfated in position 4 while the individual disaccharide cleaved-off by the lyase, is sulfated in position 6 of the GalNAc moiety.

Studies dedicated to hybrid CS/DS chains derived from decorin secreted by human skin fibroblasts, showed their interaction with FGF-2, as the oligosaccharides release by chondroitin B lyase digestion[35]. For identification of the biologically active sequence, we reported upon the efficiency of our improved protocol, which targeted the structural analysis of enzyme-resistant oligosaccharides larger than standard trisulfated hexasaccharides[35]. The method was based also on CE for separating oversulfated species in off-line combination with nanoESI-QTOF-MS/MS. The heterogeneity of the oligosaccharide mixture was first demonstrated by CE with UV detection. Further, by nanoESI-QTOF-MS analysis of the CE fractions, up to 12-mer oligosaccharides with different degrees of sulfation were identified. Another interesting information emerging from the MS screening of the separated CE fractions was that the species with high molar sulfate content could be clearly separated from the non-sulfated ones, present in the CS/DS mixture released by β-elimination. For strict determination of the degree of sulfation in single GAG species and delimiting the real under- and nonsulfated species from the possible artifacts induced by the in-source decay of the sulfate groups in the MS mode, this aspect was crucial.

In analogy to the previous reports upon ESI-MS methods for GAG oligosaccharide analysis[28,29] it was observed that in the negative ESI-MS, the in-source desulfation may be reduced by acquiring the spectra under mild values of the MS counterelectrode potential. Using this procedure, it was possible to detect up to fully sulfated dodecasaccharide and a number of intact oversulfated species (Table 1).

**TABLE 1**

| m/z | Charge state | Structure |
|---|---|---|
| 458.02 | 4 | $IdoAGalNAc[GlcAGalNAc]_3(4S)$ * |
| 511.38 | 3 | $IdoAGalNAc[GlcAGalNAc]_2(5S)$ ** |
| 517.38 | 3 | $IdoAGalNAc[GlcAGalNAc]_2(5S)$ ** |
| 533.88 | 5 | $IdoAGalNAc[GlcAGalNAc]_5$ (5S) |
| 552.66 | 4 | $IdoAGalNAc[GlcAGalNAc]_4$ (4S) |
| 572.63 | 4 | $IdoAGalNAc[GlcAGalNAc]_4$ (5S) * |
| 585.03 | 3 | $IdoAGalNAc[GlcAGalNAc]_3$ (3S) |
| 611.28 | 3 | $IdoAGalNAc[GlcAGalNAc]_3(4S)$ * |
| 647.40 | 4 | $IdoAGalNAc[GlcAGalNAc]_5$ (4S) |
| 664.89 | 3 | $IdoAGalNAc[GlcAGalNAc]_3(6S)$ ** |
| 687.38 | 2 | $IdoAGalNAc[GlcAGalNAc]_2(3S)$ * |
| 711.89 | 3 | $IdoAGalNAc[GlcAGalNAc]_4$ (3S) * |
| 764.03 | 3 | $IdoAGalNAc[GlcAGalNAc]_4(5S)$ * |
| 877.25 | 2 | $IdoAGalNAc[GlcAGalNAc]_3$ (3S) |
| 917.13 | 3 | $IdoAGalNAc[GlcAGalNAc]_5(6S)$ * |

*Fully sulfated species
**Oversulfated species

Detailed structural characterization was achieved by fragmentation of the novel CS/DS-containing hexasaccharide, 4,5-Δ-$IdoAGalNAc[GlcAGalNAc]_2(5S)$, for determination of the sulfation pattern along the carbohydrate chain. According to the MS/MS data three sulfates were found distributed in the IdoAGalNAcGlcA moiety, offering two structural motifs: one containing the sulfated L-IdoA and the disulfation of GalNAc moiety, and the other with the both UroA moieties and the GalNAc each monosulfated (Fig.1). The structural data gathered for the novel oligosaccharide 4,5-Δ-$IdoAGalNAc[GlcAGalNAc]_2(5S)$ confirmed the presence of a tetrasulfated tetrasaccharide partial sequence assigned either to the IdoA(S)GalNAc(S)GlcA(S)GalNAc(S) or to the IdoA(S)GalNAc(2S)GlcAGalNAc(S) moiety.

In our recent study, the hybrid CS/DS chains derived from decorin secreted by human skin fibroblasts were also released by β-elimination and further subjected to depolymerization using chondroitin AC lyase. Following the procedure we described previously[35] the resulting oligosaccharide mixture was separated by SEC. The octasaccharide fraction was collected, purified and analyzed by advanced, highly sensitive chip electrospray ionization using fully automated sample infusion into QTOF-MS. The chip nanoESI-MS revealed hexa-, penta and quadruply charged molecular ions corresponding to the unsaturated regularly sulfated DS octasaccharide having the composition of 4,5-Δ-$GlcAGalNAc[IdoAGalNAc]_3(4S)$, and a triply charged ion assigned to an undersulfated octasaccharide motif 4,5-Δ- $GlcAGalNAc[IdoAGalNAc]_3(3S)$ (Fig.2). The oligosaccharide 4,5-Δ-$GlcAGalNAc[IdoGalNAc]_3(4S)$ could be part of the structure for activation heparin cofactor II where

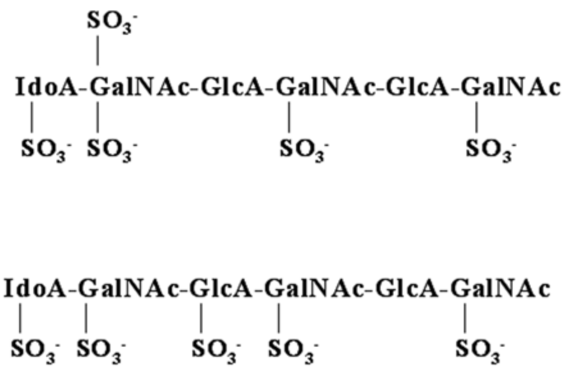

**FIGURE 1.** Two alternative structure proposals for the pentasulfated CS/DS unsaturated hexasaccharide 4,5-Δ-IdoAGalNAc[GlcAGalNAc]$_2$(5S) from human skin fibroblast decorin. The upper structure proposal is the more probable one. Adapted and reprinted with permission from [35].

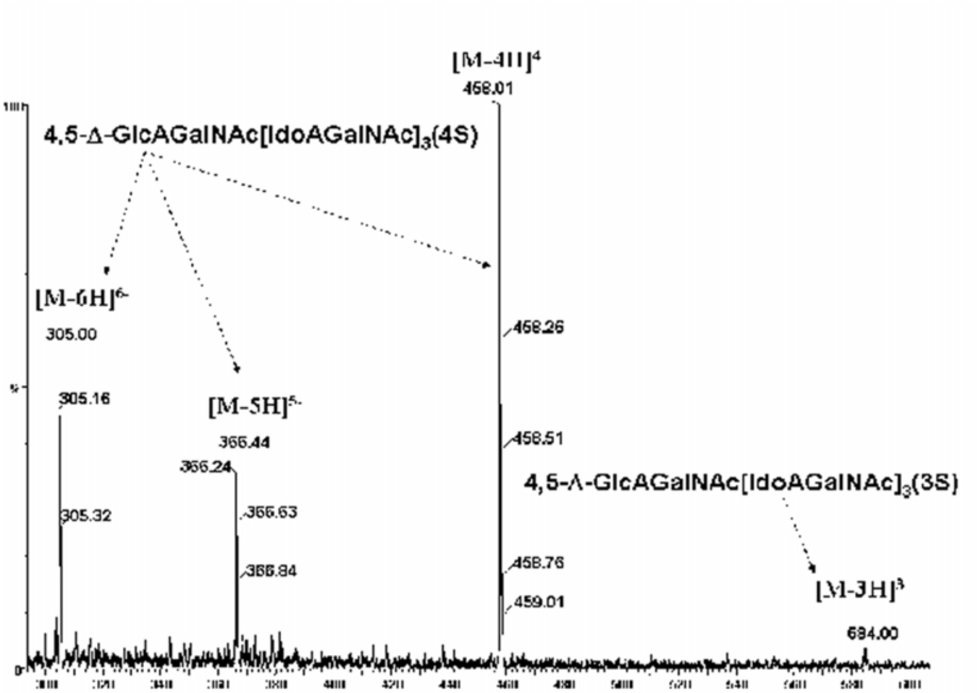

**FIGURE 2.** Negative ion mode chip nanoESI QTOF mass spectrum of the DS octasaccharide fraction released from human skin fibroblasts decorin by β-elimination and deploymerization with chondroitin AC lyase. The octasaccharide fraction was separated by SEC and purified using Sephadex G25 prior to chip MS analysis.

analysis of the hexasaccharide showed that it is composed of disaccharides units: IdoA(S)-GalNAc(S) with IdoA sulfated in position 2 and GalNAc in position 4 and GlcA-GalNAc(2S) with GalNAc sulfated in position 4 and 6[36].

With neoglycolipid microarrays Yamaguchi and co-workers[37] showed that DS-oligosaccharides larger than octamers bind preferentially FGF-7, HGF and heparin cofactor II. These results show the

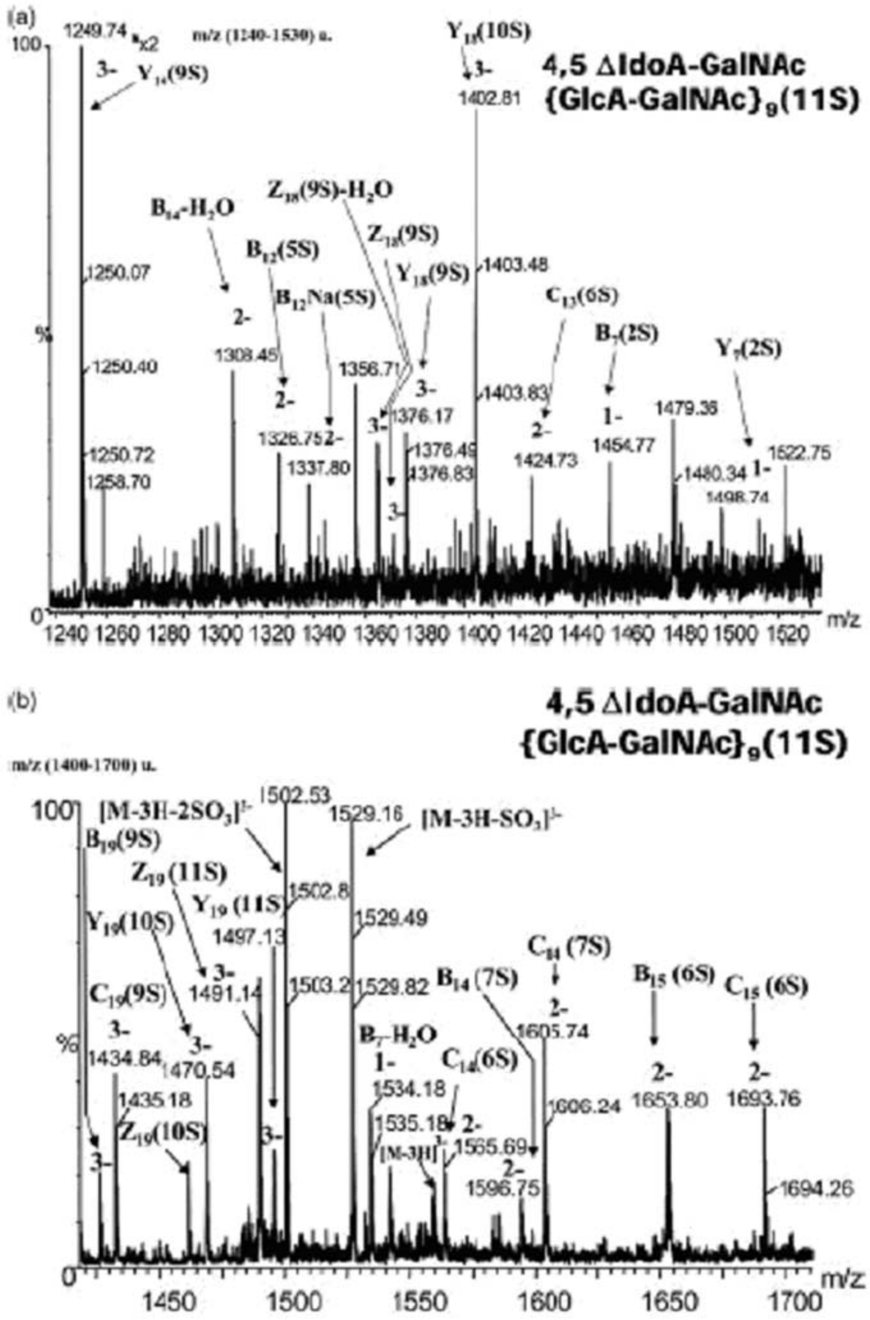

**FIGURE 3** Sheathless on-line CE/negative ion mode nanoESI-QTOF-auto MS/MS of the oversulfated unsaturated eicosasaccharide 4,5-Δ-IdoAGalNAc[GlcAGalNAc]$_9$(11S) detected as a pentacharged ion at *m/z* 933.12. (a) *m/z* (1240–1530) u; (b) *m/z* range (1400–1700). Assignment of fragment ions is according to Domon and Costello [39]. Reprinted with permission from [38].

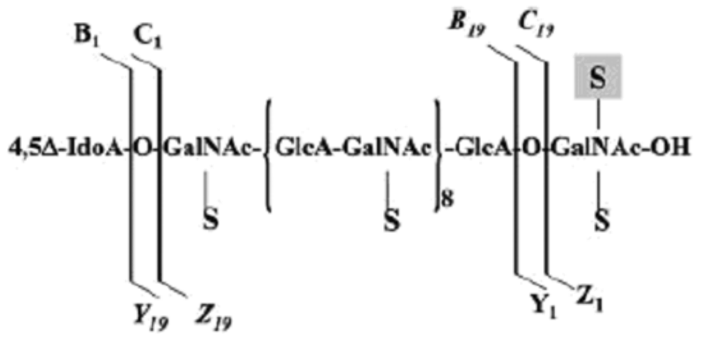

**FIGURE 4.** Structural proposal for the oversulfated unsaturated eicosasaccharide 4,5-Δ-IdoAGalNAc[GlcAGalNAc]$_9$(11S) identified in galactosaminoglycans mixture derived from recombinant expressed decorin according to the data obtained by on-line CE/ESI-autoMS/MS. Reprinted with permission from [38].

importance of sequencing longer galactosaminoglycan chains to determine the binding and active structure. Reliable data upon extended galactosaminoglycan chains[38] in mixtures from conditioned culture medium of 293 cells transfected with cDNA for decorin, containg mainly CS structures [11], were obtained using a novel approach based on on-line CE/ESI-QTOF-MS and tandem MS. An unsaturated oversulfated tetradecasaccharide assigned to the composition of 4,5-Δ-IdoAGalNAc[GlcAGalNAc]$_6$(9S), a saturated eicosasaccharide IdoAGalNAc[GlcAGalNAc]$_9$(11S) an unsaturated octadecasaccharide 4,5-Δ-IdoAGalNAc[GlcAGalNAc]$_8$(10S) and an abundant unsaturated eicosasaccharide bearing eleven sulfate groups having the structure of 4,5-Δ-IdoAGalNAc[GlcAGalNAc]$_9$(11S) were identified. These data demonstrated the complexity of the sample, which is related to the elevated length of the GAG oligosaccharide chains present, as well as their type and high level of sulfation. The molecular ions obtained in this experiment were shown to carry different numbers of sulfate groups per disaccharide unit and were all expressing oversulfation of the molecules. High percentage of species separated and detected in the mass spectra was observed to carry one double bond, originating from the specific eliminative action of chondroitin B lyase on GalNAc-IdoA linkages. The structural information upon the oversulfated unsaturated eicosasaccharide 4,5-Δ IdoAGalNAc [GlcAGalNAc]$_9$(11S) were acquired by the advanced data-dependent MS method. In the MS to MS/MS switching approach applied to the pentacharged 4,5-ΔIdoAGalNAc[GlcAGalNAc]$_9$(11S) (Fig. 3) the product ion analysis generated fair fingerprint ion set for identification of the molecular structure in terms of epimerization and sulfation pattern (Fig. 4). According to this diagnostic group of ions and the rest of regularly and oversulfated fragments, the additional sulfate group is indubitably linked to the last GalNAc moiety at the reducing end.

All these structural studies demonstrate in the first place that the determination of molecular characteristics of CS/DS, is an essential prerequisite in understanding their functions. To understand the functional significance of CS/DS one must consider both the structure of the CS/DS chain and the core protein, in this case decorin protein core, which leads the GAG chain into the extracellular matrix. Secondly, these findings show that the structural analysis must start with the identification of all glycoforms in complex mixtures obtained after detachment from core-protein by chemical or enzymatic methods followed by collecting and assembling detailed data upon epimerization, sulfate content and its distribution. Lastly, it appears clear that the presence of regular or irregular domains plays a critical biological role and can be directly recognized only by sequencing single components in tandem MS experiments.

## ACKNOWLEDGEMENTS

This work has been carried out within the Sonderforschungsbereich (SFB) 492 „Extracellular Matrix: Biogenesis, Assembly and Cellular Interaction"(Projects A9) of the Deutsche Forschungsgemeinschaft to D.G.S. and Research of Excellence grants CE.Ex. 14/2005 and 98/2006 provided by the Romanian Ministry of Education and Research to A.D.Z.

## References

1. Iozzo, R.V. (1998) Matrix proteoglycans: from molecular design to cellular function. *Annu. Rev. Biochem.* **67**, 609-652.
2. Kresse, H., Rosthoj, S., Quentin, E., Hollmann, J., Glössl, J., Okada, S. and Tonnesen, T. (1987) Glycosaminoglycan-free small proteoglycan core protein is secreted by fibroblasts from a patient with a syndrome resembling progeroid. *Am. J. Hum. Genet.* **41**, 436-453.
3. Faiyaz-Ul-Haque, M., Zaidi, S.H., Al Ali, M., Al Mureikhi, M.S., Kennedy, S., Al Thani, G., Tsui, L.C., and Teebi, A.S. (2004) A novel missense mutation in the galactosyltransferase-I (B4GALT7) gene in a family exhibiting facioskeletal anomalies and Ehlers-Danlos syndrome resembling the progeroid type. *Am. J. Med. Genet.* **128**A, 39-45.
4. Seidler, D.G., Faiyaz-Ul-Haque, M., Hansen, U., Yip, G.W., Zaidi, S.H.E., Teebi, A.S., Kiesel, L. and Götte, M. (2006) Defective glycosylation of decorin and biglycan, altered collagen structure and abnormal phenotype of the skin fibroblasts of an Ehlers-Danlos syndrome patient carrying the novel Arg270Cys substitution in galactosyltransferase I (ß4GalT-7). *J. Mol. Med.* **84**, 584-584.
5. Danielson, K.G., Baribault, H., Holmes, D.F., Graham, H., Kadler, K.E., and Iozzo, R.V. (1997) Targeted disruption of decorin leads to abnormal collagen fibril morphology and skin fragility. *J. Cell Biol*. **136**, 729-743.
6. Xu, T., Bianco, P., Fisher, L.W., Longenecker, G., Smith, E., Goldstein, S., Bonadio, J., Boskey, A., Heegaard, A.M., Sommer, B., Satomura, K., Dominguez, P., Zhao, C., Kulkarni, A.B., Robey, P.G., and Young, M.F. (1998) Targeted disruption of the biglycan gene leads to an osteoporosis-like phenotype in mice. *Nat. Genet.* **20**, 78-82
7. Corsi, A., Xu, T., Chen, X.D., Boyde, A., Liang, J., Mankani, M., Sommer, B., Iozzo, R.V., Eichstetter, I., Robey, P.G., Bianco, P., and Young, M.F. (2002) Phenotypic effects of biglycan deficiency are linked to collagen fibril abnormalities, are synergized by decorin deficiency, and mimic Ehlers-Danlos-like changes in bone and other connective tissues. *J. Bone Miner. Res.* **17**, 1180-1189.
8. Ferro, D.R., Provasoli, A., Ragazzi, M., Casu, B., Torri, G., Bossennec, V., Perly, B., Sinay, P., Petitou, M., and Choay, J.(1990) Conformer populations of L-iduronic acid residues in glycosaminoglycan sequences. *Carbohydr. Res.*, **195**, 157-167.
9. Maccarana, M., Olander, B., Malmström, J., Tiedemann, K., Aebersold, R., Lindahl, U., Li, J.P. and Malmström, A. (2006) Biosynthesis of dermatan sulfate: chondroitin-glucuronate C5-epimerase is identical to SART2. *J. Biol. Chem.* **281**, 11560-8.
10. Nakao, M., Shichijo, S., Imaizumi, T., Inoue, Y., Matsunaga, K., Yamada, A., Kikuchi, M., Tsuda, N., Ohta, K., Takamori, S., Yamana, H., Fujita, H., and Itoh, K. (2000) Identification of a gene coding for a new squamous cell carcinoma antigen recognized by the CTL. *J. Immunol.* **164**, 2565-2574.
11. Seidler, D.G., Breuer, E., Grande-Allen, K.J., Hascall, V.C., and Kresse, H. (2002) Core protein dependence of epimerization of glucuronosyl residues in galactosaminoglycans. *J. Biol. Chem.* **277**, 42409-42416.
12. Bernfield, M., Götte, M., Park, P.W., Reizes, O., Fitzgerald, M.L., Lincecum, J. and Zako, M. (1999) Functions of cell surface heparan sulfate proteoglycans. *Ann. Rev. Biochem.* **68**, 729-777.
13. Lee, P.H., Trowbridge, J.M., Taylor, K.R., Morhenn, V.B. and Gallo, R.L. (2004) Dermatan sulfate proteoglycan and glycosaminoglycan synthesis is induced in fibroblasts by transfer to a three-dimensional extracellular environment. *J. Biol. Chem.* **279**, 48640-48646.
14. Mascellani, G., Liverani, L., Bianchini, P., Parma, B., Torri, G., Bisio, A., Guerrini, M. and Casu, B. (1993) *Biochem. J.* **296**, 639-648.
15. Priglinger, U., Geiger, M., Bielek, E., Vanyek, E. and Binder, B.R. (1994) Binding of urinary protein C inhibitor to cultured human epithelial kidney tumor cells (TCL-598). The role of glycosaminoglycans present on the luminal cell surface. *J. Biol. Chem.*, **269**, 14705-14710.
16. Walker, A. and Gallagher, J.T. (1996) Structural domains of heparan sulphate for specific recognition of the C-terminal heparin-binding domain of human plasma fibronectin (HEPII). *Biochem. J.* **317**, 871-817.
17. Cella, G., Boeri, G., Saggiorato, G., Paolini, R., Luzzatto, G. and Terribile, V.I. (1992) Interaction between histidine-rich glycoprotein and platelet factor 4 with dermatan sulfate and low-molecular-weight dermatan sulfate. *Angiology* **43**, 59-62.

18. Denholm, E.M., Lin, Y.Q. and Silver, P.J. (2001) Anti-tumor activities of chondroitinase AC and chondroitinase B: inhibition of angiogenesis, proliferation and invasion. *Eur. J. Pharmacol.* **416**, 213-221.
19. Kawashima, H., Atarashi, K., Hirose, M., Hirose, J., Yamada, S., Sugahara, K. and Miyasaka, M. (2002) Oversulfated chondroitin/dermatan sulfates containing GlcAbeta1/IdoAalpha1-3GalNAc (4, 6-O-disulfate) interact with L- and P- selectin and chemokines. *J. Biol. Chem.* **277**, 12921-12930.
20. Vlodavsky, I., Folkman, J., Sullivan, R., Fridman, R., Ishai-Michaeli, R., Sasse, J., Klagsbrun, M. (1987) Endothelial cell-derived basic fibroblast growth factor: synthesis and deposition into subendothelial extracellular matrix. *Proc. Natl. Acad. Sc.i U. S.A.* **84**, 2292-2296.
21. Klagsbrun, M., Baird, A. (1991) A dual receptor system is required for basic fibroblast growth factor activity. *Cell* **67**, 229-231.
22. Esko, J.D., Selleck, S.B. (2002) Order out of chaos: assembly of ligand binding sites in heparan sulfate. *Annu. Rev. Biochem.* **71**, 435-471.
23. Penc, S.F., Pomahac, B., Winkler, T., Dorschner, R.A., Eriksson, E., Herndon, M. and Gallo, R.L. (1998) Dermatan sulfate released after injury is a potent promoter of fibroblast growth factor-2 function. *J. Biol. Chem.* **273**, 28116-28121.
24. Jarvelainen, H., Puolakkainen, P., Pakkanen, S., Brown, E.L., Hook, M., Iozzo, R.V., Sage, E.H., Wight, T.N. (2006) A role for decorin in cutaneous wound healing and angiogenesis. *Wound Repair Regen.* 14, 443-452.
25. Taylor, K.R., Rudisill, J.A. and Gallo, R.L. (2005) Structural and sequence motifs in dermatan sulfate for promoting fibroblast growth factor-2 (FGF-2) and FGF-7 activity. *J. Biol. Chem.* **280**, 5300-5306.
26. Lyon, M., Deakin, J.A., Rahmoune, H., Fernig, D.G., Nakamura, T. and Gallagher, J.T. (1998) Hepatocyte growth factor/scatter factor binds with high affinity to dermatan sulfate. *J. Biol. Chem.* **273,** 271-278.
27. Desnoyers, L., Arnott, D. and Pennica, D. (2001) WISP-1 binds to decorin and biglycan. *J. Biol. Chem.* **276**, 47599-47607.
28. Zaia, J. (2004) Mass spectrometry of oligosaccharides. *Mass Spectrom. Rev.* **23**, 161-227.
29. Miller, M.J., Costello, C.E., Malmstrom, A. and Zaia, J. (2006) A tandem mass spectrometric approach to determination of chondroitin/dermatan sulfate oligosaccharide glycoforms. *Glycobiology* **16**, 502-513.
30. Desaire, H. and Leary, J.A. (2000) Detection and quantification of the sulfated disaccharides in chondroitin sulfate by electrospray tandem mass spectrometry. *J. Am. Soc. Mass Spectrom.* **11**, 916-920.
31. Henriksen, J., Hoffmeyer Ringborg, L. and Roepstorff, P. (2004) On-line size-exclusion chromatography/massspectrometry of low molecular mass heparin *J. Mass Spectrom.* **39**, 1305–1312.
32. Hitchcock, A.M., Costello, C.E. and Zaia, J. (2006) Glycoform quantification of chondroitin/dermatan sulfate using a liquid chromatography-tandem mass spectrometry platform. *Biochemistry* **45,** 2350-2361.
33. Zamfir, A. and Peter-Katalinić, J. (2004) Capillary electrophoresis-mass spectrometry for glycoscreening in biomedical research. *Electrophoresis* **25**, 1949-1963.
34. Zamfir, A., Seidler, D., H. Kresse, H. and Peter-Katalinić, J. (2002) Structural characterization of chondroitin/dermatan sulfate oligosaccharides from bovine aorta by capillary electrophoresis and electrospray ionization quadrupole time-of-flight tandem mass spectrometry. *Rapid Commun. Mass Spectrom.* **16**, 2015-2024.
35. Zamfir, A., Seidler, D.G., Kresse, H. and Peter-Katalinić, J. (2003) Structural investigation of chondroitin/dermatan sulfate oligosaccharides from human skin fibroblast decorin. *Glycobiology* **13**, 733-742.
36. Denti, A., Sini, P., Tira, M.E. and Balduini, C. (1995) Structural heterogeneity of dermatan sulfate chains: correlation with heparin cofactor II activating properties. *Thromb. Res.* **79**, 187-198.
37. Yamaguchi, K., Tamaki, H. and Fukui, S. (2006) Detection of oligosaccharide ligands for Hepatocyte growth factor/Scatter factor (HGF/SF), Keratinocyte growth factor (KGF/FGF-7), RANTES and Heparin cofactor II by neoglycolipid microarrays of glycosaminoglycan-derived oligosaccharide fragments. *Glycoconjugate. J.* **23**, 513-523.
38. Zamfir, A., Seidler, D.G., Schönherr, E., Kresse, H., and Peter-Katalinić, J. (2004) On-line sheathless capillary electrophoresis/nanoelectrospray ionization-tandem mass spectrometry for the analysis of glycosaminoglycan oligosaccharides. *Electrophoresis* **25**, 2010-2016.
39. Domon, B. and Costello, C.E. (1988) A systematic nomenclature for carbohydrate fragmentations in FAB-MS/MS spectra of glycoconjugates. *Glycoconjugate J.* **5**, 397-409.
